# Rehabilitation robotics and allied digital technologies: opportunities, barriers and solutions for improving their clinical implementation. A position paper from the Fit for Medical Robotics Initiative

**DOI:** 10.3389/frobt.2025.1531067

**Published:** 2025-07-03

**Authors:** Irene Giovanna Aprile, Silvana Quaglini, Giuseppe Turchetti, Leandro Pecchia, Giovanni Comandè, Furio Gramatica, Emanuele Gruppioni, Giuseppina Sgandurra, Christian Cipriani, Fit4Mission1 group

**Affiliations:** ^1^ Neurorehabilitation Department, IRCCS Fondazione Don Carlo Gnocchi ONLUS, Florence, Italy; ^2^ Department of Electrical, Computer and Biomedical Engineering, University of Pavia, Pavia, Italy; ^3^ Institute of Management, Scuola Superiore Sant’Anna, Pisa, Italy; ^4^ School of Engineering, University of Warwick, Coventry, United Kingdom; ^5^ School of Engineering, Campus Biomedico of Rome, Rome, Italy; ^6^ Development and Innovation Department, IRCCS Fondazione Don Carlo Gnocchi ONLUS, Milan, Italy; ^7^ Research and Training Area of the INAIL Prosthetics, Centro Protesi INAIL, Istituto Nazionale Assicurazione Contro Gli Infortuni sul Lavoro, Bologna, Italy; ^8^ Department of Developmental Neuroscience, IRCCS Fondazione Stella Maris, Pisa, Italy; ^9^ Department of Clinical and Experimental Medicine, University of Pisa, Pisa, Italy; ^10^ BioRobotics Institute, Scuola Superiore Sant’Anna, Pontedera, Italy

**Keywords:** rehabilitation, robotics, digital technologies, healthcare technologies, clinical translation, pragmatic clinical trials

## Abstract

Robotics has been proposed as a promising solution for treating individuals with motor, sensory, and/or cognitive disabilities. Despite the great technological effort put into this field, the translation of robots from the laboratory to the clinical environment is not a seamless and smooth process, and their real-world adoption remains limited. Several barriers to the introduction of robotics in clinical practice have been identified, including a lack of sufficient scientific evidence about its actual cost/effectiveness, resistance to adopting these technologies, and economic, ethical, and regulatory restraints. Fit for Medical Robotics (Fit4MedRob) is an ambitious Initiative designed to bridge the gap between technological innovation and clinical application. One of the main goals of the Initiative is to conduct large-scale pragmatic trials to evaluate the effectiveness and the sustainability of commercially available robotic solutions. To guide the design of these trials, different online surveys have been implemented and delivered to identify the needs of healthcare practitioners and patients at different phases of the disease (acute to chronic) and therapeutic settings (hospital to home care). The results of the Initiative will suggest new organizational models to effectively introduce robotics-assisted rehabilitation into clinical practice. The paper will report on the opportunities of robotics for rehabilitation, the barriers to their clinical implementation, and the proposal of Fit4MedRob to overcome such limitations and facilitate the effective clinical implementation of robotic solutions.

## 1 Introduction

Robotics started to be used in rehabilitation in the late 1980s, but its development has experienced exponential growth in the last 20 years. A search on Pubmed using the keywords “(REHABILITATION) AND (ROBOT*)” in the title or abstract yields only 55 articles from 1988 to 2000, whereas from 2000 to the present, the number of articles increases to 5052.

Robots and allied digital technologies have been proposed as resources capable of revolutionizing and enhancing the efficacy of rehabilitation treatments. As a matter of fact, in traditional treatments, motor exercises are generally performed with the physical assistance of physiotherapists, leading to issues related to the availability of time and human resources, which may significantly impair treatment outcomes (Laut et al., 2016). Thus, the first rehabilitation robots were designed (i) to amplify the treatment dose (Prange et al., 2006), particularly in patients with severe motor deficits, and (ii) to alleviate the burden on physiotherapists (Riener, 2012; Laut et al., 2016). More recently, the use of robots has been extended, covering specific needs of heterogeneous pathologies and settings. Despite the growing interest of clinicians, also driven by the development of new robots, the process of transferring robots from the research laboratory to the clinical environment remains complex, likely because it requires strong collaboration among various professionals, such as doctors, engineers, and computer scientists. Rehabilitation is a field where practitioners are accustomed to working in a multidisciplinary setting involving physiatrists, neurologists, physiotherapists, occupational therapists, speech therapists, and neuropsychologists. However, the difficulty likely lies in bridging the gap between clinicians, computer scientists, and engineers. These are, in fact, different worlds that speak very different languages. In addition to the need for greater interaction among the different professional figures involved, there are other barriers that make the transfer of robotics and technologies into clinical practice complex. Among these barriers, resistance to adopting these technologies, and economic, ethical, and legal constraints should be highlighted. Last but not least, evidence on the effectiveness and sustainability of robotics-assisted rehabilitation is still poorly addressed in the scientific literature. First of all, most studies focus on post-stroke rehabilitation, with other pathologies being underrepresented. Second, the results are often contradictory (Mehrholz et al., 2018; Wu et al., 2021; Yang et al., 2023). This is due to multiple factors, and particularly to the considerable heterogeneity of studies in terms of treatment duration, session frequency, and specific treatment modalities. Indeed, the literature mainly includes exploratory trials that consider groups of patients with a specific level of disability, assessed with specific recovery metrics, and this explains the variability in trials and the data collected. Additionally, the limited number of patients treated in many studies represents a significant obstacle to the overall evaluation of effectiveness. In studies with multicenter designs and/or greater sample sizes, robot-assisted gait training (Calafiore et al., 2022; Wu et al., 2023) and upper limb treatment (Rodgers et al., 2019; Aprile et al., 2020) have been shown to be as effective as conventional therapies. In other neurological conditions, such as traumatic brain injury, cerebral palsy, and spinal cord injury, studies are scarce, and on small patient cohorts (Turolla et al., 2022).

Beyond the limited results in the scientific literature, robotics, in principle, shows real potential and seems to be a useful tool for revolutionizing rehabilitation practices and improving existing services, enabling the treatment of a larger number of patients compared to conventional rehabilitation and ensuring continuity of care. In fact, while the first robots were designed to only increase the intensity of the treatments, today’s available devices when used with specific organizational models, can allow for the treatment of more patients simultaneously. Moreover, robots also allow for the rehabilitating of both, specific motor functions (movement coordination and velocity, muscle strength, balance, walking), and cognitive functions (attention, memory, executive function), while enabling the reach of patients remotely through telerehabilitation and telerobotics.

To gain definitive scientific evidence regarding robotic-assisted rehabilitation, thus bridging the gap between potential perceived benefits and scientific evidence, we are actively working within an Initiative, called Fit for Medical Robotics (Fit4MedRob, https://www.fit4medrob.it/). Fit4MedRob is funded by the Italian Ministry of University and Research, and one of its core principles is the belief that available robotic solutions must be validated into large, well-designed pragmatic clinical trials, with an active involvement of healthcare professionals with specific skills and training in robotics. Similarly, since any new technology implies an organizational change within healthcare institutions, new organizational models for robotic-supported treatments need to be developed and tested to assess their cost-effectiveness and sustainability. Finally, it is assumed that robust regulatory frameworks, along with clear reimbursement policies, are crucial to ensure equitable access for all patients nationwide.

**FIGURE 1:**
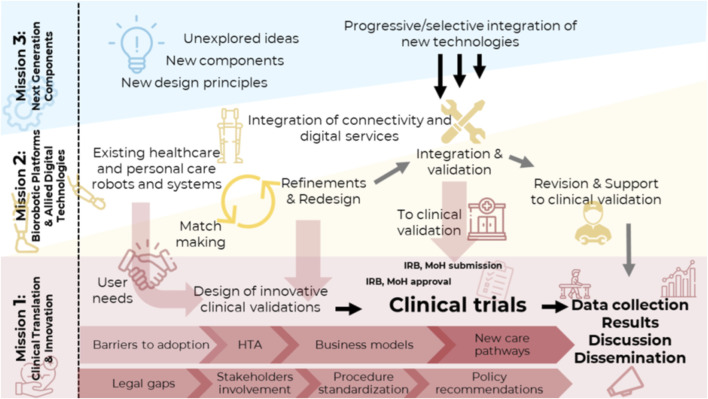
Graphical representation of the interactions and main activities within the three Missions of the Fit for Medical Robotics Initiative.

Fit4MedRob, which will last 44 months, is divided into three interconnected missions (Figure 1). The present paper focuses on Mission 1. While Mission 2 carries on explanatory trials to evaluate the efficacy of prototypical robotics devices, and Mission 3 studies innovative technologies for a future generation of rehabilitation robots, Mission 1 coordinates a set of pragmatic clinical trials to test the superiority or non-inferiority of existing, commercial robotic solutions, compared to traditional rehabilitation treatments. Mission 1 also aims at identifying both healthcare practitioners’ and patients’ needs in the various phases of an ongoing disease (from acute to chronic) and different treatment settings (from hospital to home-based care). Understanding end-user needs has proven useful in guiding the design of pragmatic large-case trials, not only for better coping with clinical needs but also for testing the above-mentioned new organizational models of treatment. Within the Fit4MedRob Initiative, different patient populations are being recruited across various trials, ranging from adult post-stroke patients to children with disabilities.

This paper will discuss the opportunities offered by robotics for rehabilitation, the barriers to its clinical implementation, and the proposal of Fit4MedRob to overcome those barriers. Section 2 will dive into the opportunities of robotics-assisted rehabilitation, while Section 3 will outline barriers and limitations for the clinical implementation of robotic technologies. Section 4 will present the solutions proposed by Fit4MedRob, and Section 5 will report an overall discussion.

## 2 Opportunities of robotics in rehabilitation

In recent decades, technological innovation has led to the development of complex systems and digital platforms aimed at meeting specific rehabilitation needs (Loureiro et al., 2011; Banyai and Brişan, 2024). Robotic therapy has been suggested as an effective method to enhance both the quantity and intensity of therapy and, when required, to standardize treatment by providing a complex but controlled multisensory stimulation (Masiero et al., 2014; Gassert and Dietz, 2018). Robotic devices aid patients in completing necessary tasks while preventing improper movements (Lum et al., 2012). Furthermore, including sensory feedback in robotic systems, such as visual and auditory stimuli, promotes brain plasticity and enhances rehabilitation outcomes (Rosati et al., 2013; Zheng et al., 2023).

One of the most important aspects of these technologies is the possibility of promoting personalized medicine. In fact, digital and robotic technologies may be programmed to match the specific needs of each patient, providing personalized treatment protocols that adapt to individual progress. To this end, robotic devices can be equipped with advanced sensors and actuator technologies, which can objectively and quantitatively measure a patient’s motor status and progress. By collecting and processing kinematic and kinetic data, devices generate numerical data that provide valuable insights into a patient’s performance (Germanotta et al., 2018). This information assists clinicians in evaluating their patients and in tailoring rehabilitation protocols to individual needs, potentially enhancing overall effectiveness. With the same intent, wearable sensors can track physiological characteristics such as muscle activity and movement patterns, providing real-time information that, again, can be used to customize rehabilitation protocols.

Treatment customization could be further improved by the incorporation of Machine Learning (ML) and Artificial Intelligence (AI) (Schork, 2019; Nicora et al., 2024b). To identify patterns and predict the best plan of action for each patient, AI algorithms can examine data collected by sensors and robotic equipment. These insights enable the development of highly individualized therapy plans that can evolve over time based on the patient’s progress and changing needs.

In addition, even if on a smaller scale with respect to the clinical setting, advanced technologies for home-based treatment were also developed, with the potential to provide a continuum of care throughout the entire chronic phase of the disease. These solutions can reduce the burden on healthcare facilities by enabling patients to participate in therapeutic activities and receive real-time feedback from the comfort of their homes. The possibility of performing therapy at home can encourage long-term commitment to treatment procedures, which is crucial for recovery and maintenance of the progress achieved (Argent et al., 2018).

Eventually, digital technologies such as virtual reality (VR) have introduced new dimensions to rehabilitation. VR environments replicate real-life scenarios, giving patients a safe, immersive environment in which to practice activities of daily living (e.g., cooking, self-caring, gardening, etc.), which helps them regain confidence and independence. The immersive nature of VR increases patient motivation and engagement, both of which are essential for positive rehabilitation outcomes (Mouatt et al., 2020).

## 3 Barriers to the clinical implementation of robotics

A growing body of literature highlights a significant limitation in the clinical adoption of advanced technologies in rehabilitation. Turchetti et al. (2014) illustrated that, although there is a positive momentum in the market, the overall penetration of robotic systems in clinical practice remains very low compared to the potential suggested by studies on clinical efficacy. Mehrholz et al. (2018) and Wu et al. (2021) report that, while robotic devices show potential in improving motor outcomes, their use is still confined primarily to research settings due to costs, lack of standardization, and insufficient evidence from large-scale pragmatic trials. Moreover, due to cost issues, disparities are evident at the geographical level. A recent survey (Johnson et al., 2023) found that rehabilitation robots are primarily available in high-income countries due to their high cost, which limits their accessibility in low- and middle-income countries. According to Boldrini et al. (2023), in Italy, the use of robotic technologies in rehabilitation, particularly for neurorehabilitation, is expected to grow; however, a shared reference framework in the healthcare system is currently missing, which complicates the structured introduction of these technologies into rehabilitation services.

**FIGURE 2:**
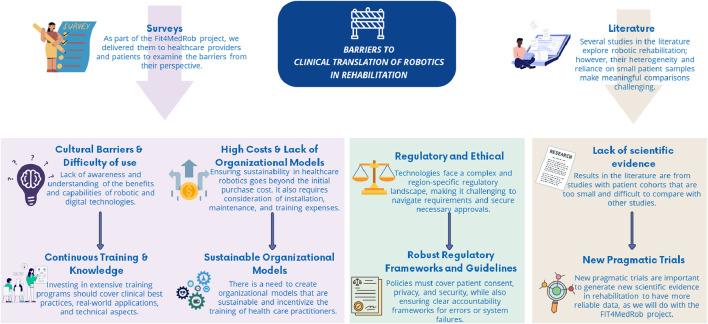
Identified barriers to the clinical translation of robotics in rehabilitation and the specific strategies to overcome them.

Therefore, despite the promising potential of robotic and allied digital technologies, multiple challenges impede their implementation in clinical settings. These barriers are complex, encompassing resistance to adopting these technologies, economic, and regulatory challenges that must be overcome by implementing specific strategies (Figure 2).

### 3.1 Sustainability issues

In order to favor the introduction and adoption of robotic solutions in rehabilitation procedures, it is necessary to demonstrate - from a Health Technology Assessment (HTA) perspective (Turchetti et al., 2010) - their value with respect to the economic, organizational, usability/acceptability, ethical/legal implications, and to design business models that are sustainable both for technology producers and for technology adopters. The implementation of robotics in healthcare settings poses an economic challenge in terms of maintaining a sustainable environment (Turchetti et al., 2014). Currently, there is a scarcity of studies that have adequately and thoroughly examined the cost-effectiveness of robots in rehabilitation (Wagner et al., 2011; Gower et al., 2024). This lack of data has hampered the possibility of specific legislation governing the use of robotics in the rehabilitation field, including the reimbursement of robotic rehabilitation sessions as distinct from conventional rehabilitation sessions, and more informed decision-making by both payers and technology adopters.

In terms of economic dimension, the models to be employed are the cost-effectiveness, the cost-utility, and the cost-benefit models. They compare the new approach that adopts robotic technologies with the standard approach, considering in the analysis both the costs and benefits of the different alternatives. To conduct these analyses, several costs must be considered using a micro-costing approach, such as:

- *Direct healthcare costs*:
  • Unit cost of the technology per session (including the cost of the technology, depreciation rates based on the estimated number of treated patients when centers are fully operational, maintenance costs, energy consumption, consumables, etc.);
  • Unit cost of staff involved in rehabilitation sessions;
  • Hospitalization costs.
- *Direct non-healthcare costs*, such as expenses not reimbursed by the health service but necessary to manage the patients, like transportation costs to reach the rehabilitation center, etc.
- *Indirect costs* associated with the loss of productivity for the patient and/or their family members assisting during working hours.

These analyses are useful for: a) demonstrating that the proposed solutions are sustainable and can be reimbursed by the payers (data reported in the dossiers by technology producers have to be present for obtaining the reimbursability of the proposed healthcare solutions, and thus for increasing the adoption and diffusion of robotic health technologies into the market); b) supporting potential adopters, such as healthcare providers, to evaluate whether the adoption and use of the developed and ready-to-the-market robotic solutions produce economic and organizational benefits in their institution, given their organizational, economic and financial structure.

A comparison of conventional and robotic rehabilitation reveals that the economic dimension is strictly connected to the organizational one. From the perspective of the healthcare organization, if a physical therapist treats one patient in traditional rehabilitation, a single therapist trained in robotics could treat more patients simultaneously in one single session using robotics and technologies (Mazzoleni et al., 2014; Aprile et al., 2019; Gower et al., 2024). Still, these new organizational models need to be tested and validated, to provide sound cost-effectiveness figures of robotic technology compared to traditional approaches. Costs of these technologies encompass not only the purchase of equipment but also expenses for installation, maintenance, and training to guarantee adequate use by the clinical personnel. Thus, costs are highly variable and may range from a few hundred euros to hundreds of thousands of euros.

Nowadays, reimbursement and funding mechanisms may not always fully cover the costs associated with these technologies, further restricting their availability and accessibility. Therefore, it is necessary to design new organizational models that can ensure the effectiveness and sustainability of treatment. By demonstrating the long-term benefits of robotic and digital technologies through rigorous cost-effectiveness and cost-utility analyses, the value of these innovations can be better understood.

### 3.2 Resistance to adoption by healthcare professionals

Sustainable organizational models of treatment can be facilitated by establishing dedicated units or teams within healthcare facilities, to focus on the use of robotic and digital systems. These units may play an important cultural role, by coordinating the implementation of the new technologies, providing training and support, and ensuring that technological solutions are seamlessly integrated into clinical practice. Research indicates that successful integration often requires a structured approach, including the establishment and training of an interdisciplinary team, consisting of clinicians, engineers, and computer scientists, who must work according to a clear implementation plan (Borges do Nascimento et al., 2023).

Indeed, learning and training are two important factors that influence the use and disuse of technology by healthcare professionals (Andrade et al., 2014; Mortenson et al., 2022). Investing in comprehensive training programs is therefore essential to overcome resistance to adopting these technologies. Many healthcare professionals and institutions are deeply rooted in traditional rehabilitation methods and may be reluctant to adopt new tools due to concerns about their safety, reliability, effectiveness, and sustainability. This resistance is often exacerbated by a lack of awareness and understanding of the advantages and capabilities of robotic and digital technologies. Research shows that, despite their potential benefits, many practitioners continue to use traditional methods due to unfamiliarity with new technologies and uncertainty about their effectiveness (Borges do Nascimento et al., 2023). Indeed, in a recent study, we describe the results of our surveys, which showed that healthcare professionals’ positive feelings towards robotics are correlated with actual usage of the technology (Nicora et al., 2024a). Successful integration of robotic and digital technologies requires healthcare professionals to be well-versed in the technical and practical aspects of these tools. This gap in knowledge can hinder the effective implementation and utilization of advanced rehabilitation tools.

### 3.3 Regulatory issues

Regulatory and ethical factors are also pivotal in the adoption of robotic and digital technologies. The regulatory environment for these technologies is complex all along their lifetime cycle. Since the design phase, modern robotic devices are requested to follow a wide range of legal rules to ensure safety during trial and at deployment (e.g., Regulation EU No 536/2014 of the European Parliament and of the Council of 16 April 2014 on clinical trials on medicinal products for human use, and repealing Directive 2001/20/EC; Regulation EU 2017/745 on medical devices - MDR - and Regulation EU 2017/746 on *in vitro* diagnostic medical devices). In addition, if entrenched with digital technologies and/or embedding AI components, the number, and intricacy of compliance needs increase exponentially (see the Regulation EU 2024/1689 laying down harmonized rules on artificial intelligence, the so-called AI Act). Their digital components and potential internet connection expand further the compliance needs from design to deployment in the field of cybersecurity. This complex set of legislation is not intended to hinder the development of robotics but to ensure that, if and when deployed, the robotic solution is safe and effective for patients and their caregivers. Still, it increases the costs of design and development, which in turn affects their deployment and uptake. While a high level of safety and effectiveness is obviously welcomed by all players, it still imposes a cost that end users or third-party payers need to sustain. Nevertheless, the deployment phase and its financial schemas present their regulatory conundrums as well. If we consider, as an example, the uptake of robotic solutions in Italy, we find that the set of legal rules varies across different Regions due to the shared competencies of the central State and the Regions. In addition, the regulatory framework for robotics and allied digital healthcare technologies is relatively new and open to changes. It also lacks a clear set of best practices to be followed. Relative novelty (e.g., the adoption of the AI Act), uncompleted implementation at national and local levels, cultural inconsistencies in applying existing legislations (e.g., a different reading of harmonized rules such as the data protection regulation with reference to the possible scope of consent in personal data processing, for instance), complicate the process for developers and healthcare providers to adhere to the regulatory framework. While it is essential to ensure that these technologies meet rigorous safety and regulatory requirements, it is also essential that the compliance needs are integrated as opportunities for their effective integration into clinical practice and not only as costs.

The mentioned regulatory issues hold true in Italy, even if robotic rehabilitation has been recognized as an essential part of patient care. Indeed, since the 2024 update to the Essential Levels of Care (LEA) in Italy, these technologies are included in the specialized rehabilitative services provided by the National Healthcare System under code 93.11.G: “Motor re-education using high-tech robotic assistance devices”. However, again, the general high-level legal rule (*Motor re-education using high-tech robotic assistance devices is an essential level of care that must be reimbursed by the healthcare system*). It includes specific reimbursement for robotic systems only for the outpatient setting, as above mentioned, not based on a specific economic analysis. In clinical settings, robotic rehabilitation sessions are not recognized with an *ad hoc* code: this means they are reimbursed by the Regions as third-party payers as they were conventional sessions. This has a considerable economic impact on the institutions and companies that provide these advanced rehabilitation services to patients, given the technology’s costs, and limits the possibilities of healthcare providers. Moreover, this precludes access to robotic rehabilitation by patients who cannot afford them privately. Thus, a regulatory gap becomes a roadblocker for robotic uptake, a hindrance to the protection of the health of patients and citizens, and the substantial equality principle results seriously undermined.

What we claim here is that, since the regulatory framework can only be defined after strong evidence of the effectiveness of robotic solutions, the regulatory gap and the scientific evidence gap create a deadlock. Our key claim is that large pragmatic trials are at least helpful to start overcoming such a deadlock. As an expected outcome from Fit4MedRob, we hope to contribute to a framework capable of ensuring the reimbursement and entitlement of robotic rehabilitation technologies for all patients who could medically benefit from them. This framework would help integrate effectively these technologies into the LEA enabling reimbursement policies based on economic data derived from those pragmatic trials. This approach would not only standardize access to advanced rehabilitative care but also ensure equitable distribution of resources, thus enhancing overall patient outcomes and equal treatment.

Last but not least, the use of digital technologies and wearable sensors, which often are part of robotic solutions, involves handling a significant amount of sensitive patient information if compared to traditional treatment. Personal data are needed both at the development phase, especially to train AI systems to be embedded in the robotic devices, and at the deployment level when actual patient data will drive in practice the use of robotics solutions. The existing data protection legal rules, the General Data Protection Regulation (GDPR) to begin with, aim to facilitate the circulation of personal data as long as the risks to fundamental rights deriving from personal data processing are properly addressed. This means an important effort in terms of compliance, too often perceived as a mere burden and a roadblock to research and innovation. While the hurdles posed cannot be underestimated, it is important to cast the duty to implement robust data protection measures to secure patient information and comply with relevant regulations, such as the GDPR, in the proper light: requirements are imposed to protect fundamental rights and liberties but they can be leveraged to better design the robotic solution and gain actual access to data. Indeed, robust personal data protection legislation is paramount in harnessing the power of data altruism as defined by the Data Governance Act (Regulation EU 2022/868 of the European Parliament and of the Council of 30 May 2022 on European data governance and amending Regulation EU 2018/1724). Similarly, the advanced encryption methods used to protect personal data can and must pave the way to ease the deployment of the solutions. Regular security audits, and training of all staff in data privacy practices, enhance trust and strengthen uptake. It is clear that transparency with patients about how their data is used, stored, and shared, and providing them with clear consent forms and privacy notices (Kruse et al., 2017) is a cost. But these costs can be turned into rewarded investments since these constraints help balance technological advancements with strict ethical and regulatory standards, thus building trust and ensuring the responsible deployment of these innovative technologies.

### 3.4 Overcoming the barriers

Effectively addressing those barriers requires a comprehensive approach that includes not only overcoming resistance to adopting these technologies, as well as economic, cultural, and regulatory challenges but also addressing gaps in scientific evidence. Although robotic technologies have shown promise, insufficient scientific evidence on the effectiveness of robotics in rehabilitation is present in the literature. Therapeutic interventions that are undertaken and tested under ideal conditions (e.g., in *explanatory* clinical trials that quantify *efficacy*, often in a laboratory setting) may have little effect on patients’ care in real life (hospital wards or home setting), which is measured by *pragmatic* clinical trials that assess *effectiveness*. In the ideal conditions of efficacy trials, work is conducted utilizing very diluted times that are influenced and chosen based on the study design. On the contrary, in pragmatic trials, treatment is provided and evaluated under conditions that closely resemble real clinical practice, i.e., on patients representative of the true patient populations, having these trials less restrictive inclusion criteria, and in relation to the actual timing and organization of a hospital ward (Rodgers et al., 2019); hence, the clinical study must adapt to the reality in which the treatments are administered. However, much of the research has been so far conducted on a small and highly selective group (in an *ideal world*), whereas the results on the use of robots in the *real world*, through pragmatic clinical trials, appear to be very scarce. In relation to this last limitation, some considerations should be made:

• there is no real gold standard in the field of rehabilitation; the effects of robotics in rehabilitation are frequently compared with “usual care”, an umbrella term that encompasses heterogeneous, non-objectifiable, and non-standardized conventional rehabilitation programs that are summarily described or not described at all;
• in the pursuit of methodological rigor, robotic treatments are frequently compared with the same amount (dose) of usual care, obscuring one of the most important advantages of robotics, i.e., the possibility to increase the rehabilitation practice time (in particular in the home setting);
• almost all of the studies focused primarily on motor aspects, omitting possible beneficial effects on other domains, such as sensory and cognitive domains;
• interventions did not take into consideration patients’ characteristics that could influence the clinical outcome.

All of the previously mentioned factors may help explain the literature’s findings, which suggest that, while robotics is effective, it is not superior to traditional rehabilitation methods. Recent studies, in particular, highlight the importance of adapting robotic rehabilitation to the individual needs and characteristics (motor, sensory, cognitive functions, emotional aspects, activity limitations, or participation restrictions imposed by the disability), as well as to environmental factors, according to the International Classification of Functioning, Disability, and Health (ICF) framework (World Health Organization, 2001). This could reveal subpopulations where robotics may be more effective.

Furthermore, most trials on robotic rehabilitation explore a single phase of the disease, either subacute or chronic, and lack long-term follow-up. Moreover, experiences with using robots at the patient’s home are very limited.

To evaluate and implement robotic and digital technologies in healthcare, Fit4MedRob designed pragmatic clinical trials involving large sample sizes. These trials will monitor some patients across the entire course of the disease, from the acute to the chronic phase. Indeed, such robotic and technological approaches should not be limited to clinical settings but extended to home environments as well, ensuring a continuum of care. Hence, these trials can provide comprehensive data on the real-world effectiveness and sustainability of these technologies, thereby facilitating their integration into clinical practice and home-based settings. The smooth execution of such a huge number of trials is facilitated by the fact that, among the “Centers of Excellence” that are established thanks to the Fit4MedRob Initiative, two are planned, that are specifically aimed at promoting the introduction of robotics in clinical practice. One of the tasks of these centers, together with other centers in the Consortium with specific skills in robotic rehabilitation, is to host therapists from other centers to train them on the use of the most advanced robotic devices, and to show them former experiences, overcoming their possible resistance to adopting these technologies. It is important to outline that this training is different from pure technical training about the usage of robotic devices (something that is normally delivered by the device manufacturer or vendor). It is rather a training in clinical setting aimed at sharing a real-world experience on treating patients, and more specifically regarding which protocols to use based on the pathology and the severity of the individual patient’s clinical condition.

## 4 The Fit4MedRob trials

To allow the investigation of a large variety of diseases, the Initiative included in the consortium a set of clinical partners with different clinical expertise. Thus, each partner is in charge of conducting one or more trials related to the following diseases: central nervous system injuries (stroke, multiple sclerosis, traumatic brain injuries, cerebral palsy, mild cognitive impairment, Parkinson’s disease, spinal cord injuries), peripheral nervous system injuries (polyneuropathies, amyotrophic lateral sclerosis), muscular diseases (such as muscular dystrophies), amputees and post-surgery oncological patients. Moreover, to mitigate the risk of not reaching the required sample size, cascade calls are ongoing to have additional clinical centers on board.

Before planning the trials, Mission 1 started with the identification of the specific needs of healthcare professionals, patients, and their caregivers at different stages of disease and treatment. As a matter of fact, to obtain conclusive results, pragmatic large-scale clinical trials must be designed in the light of identified users’ needs.

### 4.1 End-users needs

To design and implement effective interventions using robotics and allied digital technologies, it is fundamental to understand end-users’ needs. Matching these different needs with appropriate technology-based treatment could improve the final outcome. In Fit4MedRob the end-users include several target groups such as healthcare practitioners, patients with neuromotor diseases, workers, and frail individuals. The needs of individuals with motor, sensory, or cognitive impairments are indeed highly heterogeneous. Factors like the specific pathology, severity, and age range (childhood to elderly) can significantly influence patient’s needs. Beyond clinical conditions and phases of an ongoing disease (from acute to chronic) also the different treatment settings (from hospital to home-based care) can influence individuals’ needs. Furthermore, the current generation of robots and allied technologies may not fully meet the expectations of healthcare practitioners (as physicians, physical therapists, occupational therapists, prosthetists, nurses, etc.), preventing their translation into clinical practice. For instance, sophisticated robots are difficult to use, as they are designed without enough attention to usability. For all these reasons, the development of specific surveys aimed at understanding the needs of various target groups, including patients with neurological diseases, amputations, or oncological diseases, frail individuals and workers, as well as healthcare practitioners was imperative. To carefully capture those end-users’ needs, five different questionnaires have been administered, tailored to: (a) collaborative patients; (b) caregivers of adult patients; (c) caregivers of pediatric patients; (d) practitioners who assist patients during rehabilitation sessions; and (e) orthopedic/prosthesis technicians.

The insights gained from these surveys, i.e., the identification and analysis of these needs, have had a pivotal role in informing the planning of pragmatic clinical trials. For example, a large proportion of patients were found to have impairments across multiple ICF domains, prompting the design of trials that explore multi-domain robotic rehabilitation approaches. Regarding healthcare practitioners, training on specific devices was one of the most frequent needs, and this led to planning training sessions prior to starting the clinical studies using those devices. In fact, a comprehensive training approach is essential for ensuring healthcare professionals achieve proficiency in robotic-assisted rehabilitation. After the fundamental technical training provided by the manufacturers, specialized clinical training led by a multidisciplinary team of physicians, physiotherapists, and biomedical engineers is crucial. This training focuses on integrating clinical reasoning into robotic system use, considering both motor and cognitive aspects of patient evaluation. Key components include selecting appropriate exergames, setting robotic parameters, and ensuring optimal patient positioning and postural control, according to patient’s clinical needs. This structured approach ensures that robotic rehabilitation is tailored to patient-specific needs, optimizing therapeutic efficacy and safety.

### 4.2 The Fit4MedRob model for pragmatic clinical trials

Pragmatic clinical trials are planned to assess the clinical effectiveness and efficiency (cost-benefit) in real-world clinical practice settings. Most of the Fit4MedRob clinical trials are designed as multicenter, randomized controlled studies comparing robot-assisted therapy (using CE-marked robotic devices) with conventional rehabilitation. The trials are being conducted in hospitals, rehabilitation facilities, and home-based settings, and their pragmatic nature allows for flexibility in the choice of commercially robotic devices, although globally guaranteeing an equivalent treatment to each patient. Thus, on the one hand, all patients undergo a common protocol in terms of global treatment and class of devices used, but on the other hand, the specific interventions may be tailored based on the individual progressive recovery of the patient. Then, clinical centers may adopt robotic devices of different brands that however guarantee the training of the same function (or domain). More precisely, the robotic gym must include at least one type of robot (and/or allied technology) able to deal with the ICF domains considered in the trial. For example, centers participating in the post-stroke rehabilitation trial in clinical setting must guarantee the presence of robots for the recovery of sensory-motor abilities of the upper and lower limb (e.g., end effectors, exoskeletons), gait, balance (e.g., stabilometric platforms, treadmills, sensor-based systems) and cognitive abilities (e.g., virtual reality).

Primary and secondary endpoints include improvement in clinical outcomes (e.g., improvements in upper-limb function), neurophysiological and instrumented outcomes (e.g., movement analysis, electroencephalography), implementation metrics (e.g., therapist workload, and patient adherence), and cost analyses, the latter collected using *ad hoc* questionnaires developed by the Fit4MedRob economics team, and tailored according to the rehabilitation setting.

Inclusion criteria are designed to reflect a broad, real-world patient population. We are recruiting broad patient groups required to detect statistically significant differences in the selected outcomes. To this end, Mission 1 has established a dedicated methodological task force, responsible for overseeing the study design, ensuring methodological rigor, and providing expert guidance on sample size determination and other key design parameters. In particular, we are exploiting this big Initiative, and the close collaboration of the involved clinical centers, to collect homogeneous outcomes, overcoming the above-cited issue of heterogeneity of the existing studies. As a matter of fact, a consensus was reached among the clinical investigators on the use of a common disability outcome measure, namely the modified Barthel Index (mBI) (Shah et al., 1989). When suitable, it will represent the primary outcome of the study, otherwise, it will be included among the secondary outcomes. In particular, to avoid the floor effect of the mBI in case of trials enrolling patients with very serious disability (e.g., acquired brain injury), the mBI will be used in addition to the Disability Rating Scale (DRS) (Rappaport et al., 1982). Instead, for trials enrolling high-performance participants, the mBI will be used in addition to the World Health Organization Disability Assessment Schedule (WHODAS) scale (Federici et al., 2017) to prevent the expected ceiling effect of the mBI.

One important legacy that these clinical trials will leave is a unique, huge database of around 2000 cases, with a detailed description of patients’ profiles, disability, diary of treatments, and outcomes, which, as foreseen by the Fit4MedRob Initiative, will be made available to the scientific community, with two main goals. The first goal is the possibility of performing meta-analyses of patient data collected across different centers, made possible by the use of the common outcome measure shared by all pragmatic trials, i.e., the change in the mBI following robotics-assisted treatment, compared with the change observed in traditionally treated cohorts. The second goal is to exploit such a huge amount of data to develop AI algorithms to be integrated into robotic solutions. These algorithms could help clinicians select the best therapeutic options for the patient through decision support systems (DSS), helping personalize the rehabilitation protocol to the specific patient’s features. In fact, subpopulations could be identified who will more or less benefit from robotic solutions.

The trial with the largest sample size (596 cases) focuses on stroke, the leading cause of acquired disability in adults. These patients, also thanks to the collaboration with Stroke Units, will undergo detailed profiling across clinical, neuroimaging, neurophysiological, and biochemical-genetic aspects. This will allow the investigation of prognostic factors for post-stroke recovery. To the best of our knowledge, no previous study has simultaneously collected clinical, neuroradiological, biochemical, and genetic factors of recovery in such a large cohort of patients. These data will enable the identification of tailored rehabilitation strategies.

### 4.3 Data collection and storage

Data will be collected using REDCap (Research Electronic Data Capture), a secure, web-based software platform designed to support data capture for research studies (Harris et al., 2009; Harris et al., 2019). Additionally, a data integration platform will merge the REDCap (structured) clinical data with medical images (MRI and CT scans), physiological signals (EEG, movement sensor data, and surface electromyography), and performance metrics from selected robotic devices used during rehabilitation sessions. The platform will also provide a dashboard allowing the investigators to access a set of descriptive statistics about their ongoing trials. No sensitive data will be shared, neither in REDCap nor in the integration platform.

## 5 Discussion

Despite significant advances in the field of robotics and digital technologies, their clinical implementation in rehabilitation settings remains limited. Several factors contribute to this challenge, including the lack of robust scientific evidence in real-world settings, economic constraints, resistance to adopting new technologies, and regulatory barriers. The concepts and initiatives discussed in this paper aim to bridge these gaps and promote the integration of innovative solutions into rehabilitation practices.

In particular, literature analysis has pointed out that–among others–limiting factors to the technological adoption are: (i) lack of conclusive evidence that these technologies can actually compete with the standard of care, (ii) lack of a more comprehensive assessment of the clinical outcomes, following a robot-mediated rehabilitation path, and (iii) a demand for a more thorough assessment of the socio-economic sustainability of (p-)rehabilitation robots (both in large and smaller-scale clinical centers). To sum up these results, the main issue hindering widespread employment of robotics and digital allied technologies is the scarcity of pragmatic trials that evaluate the effectiveness and cost-effectiveness of these solutions in diverse clinical environments. Most of the existing studies focus on efficacy in controlled settings, often ignoring the complexity and constraints of real-life healthcare systems. Following the results of the online survey on patients’ needs, the pragmatic studies proposed by Fit4MedRob, across a broad spectrum of patients, treatment settings, and disease phases, will allow us to verify the effectiveness and sustainability of new treatment models that use CE-marked technologies with one of the largest samples of patients ever attempted. Indeed, even if pragmatic studies address different pathologies and different disability levels, they all share three relevant assessment scales, namely the modified Barthel Index, the Disability Rating Scale, and the WHODAS scale (as appropriate). This harmonization across trials, along with a detailed analysis of the socio-economic viability, will allow us to collect an enormous and unique dataset of disability scale outputs. In addition, the detailed profiling of the clinical, neuroimaging, neurophysiological, and biochemical-genetic aspects in subjects with stroke, which is the most common cause of acquired disability in adults and for which we will recruit 596 cases, will also allow us to identify the prognostic factors for recovery. To our knowledge, there is no data in the international literature in which all those possible predictors of recovery have been studied simultaneously in such a large series of cases.

Altogether, these features of the pragmatic trials will enable us to reach conclusive remarks on the clinical efficacy and sustainability of robot-assisted rehabilitation, with indications on how clinical models shall be revised to promote their systematic adoption. Interestingly, the same vision is being supported by a “twin” flagship Initiative of Fit4MedRob, called Swiss Neurorehab^1^ funded in Switzerland. This kind of scientific evidence—combining clinical outcomes and economic sustainability—will be pivotal for policy development and reimbursement structures, and it will be interesting to explore how the two countries will exploit the results of the clinical trials.

Regarding the sustainability of robot-assisted rehabilitation, FIT4MedRob is investigating innovative organizational models that could support it. One approach is to reorganize clinical workflows to optimize the use of robotic technologies, as demonstrated by the proposed models where one therapist can supervise multiple patients simultaneously, in a *robotic gym* with a multidomain path of technological treatment for each patient (Aprile et al., 2019; Pavan et al., 2024). Specifically, before purchasing a new technological device, the organizational model must be adapted to take into consideration a correct evaluation and adaptation of the spaces (considering the dimensions and technical characteristics of the robots), the number of patients that can be treated, possibly simultaneously, the set-up and closing times for the use of the robot (which must be adequate for the duration of the rehabilitation session), and last but not least, the choice of robots to be acquired must take into consideration the characteristics of the patients to treat, such as in case of pediatric patients.

A crucial aspect to consider in healthcare is also the balance between the costs and equality of care. The treatment of pathologies, especially chronic conditions, comes with significant financial burdens, and it is essential that everyone has the right to access treatment. However, as treatments become more sophisticated, the associated costs are growing exponentially. Most research and development of evidence-based guidelines are conducted in high-income countries with robust healthcare resources. This raises an important question: how relevant is this evidence in a global context where such resources may not exist, and where access to care must be equitable for all who need it? To address this, pragmatic studies that include thorough cost analyses—assessing the efficiency and sustainability of treatments—are necessary. The Fit4MedRob Initiative takes both aspects of accessibility and sustainability into account by designing trials that incorporate home-based care options and detailed cost evaluations. These trials aim to explore whether the integration of robotic technologies, particularly in home care settings, can broaden access to treatment, ensuring a more equitable distribution of care. By evaluating the cost-effectiveness of these innovative approaches, Fit4MedRob seeks to provide solutions that enhance both the availability and affordability of advanced rehabilitation services, thus promoting the democratization of care. In the future, from the results of the cost-effectiveness analysis of robotic rehabilitation in real-world settings, healthcare policymakers can better understand how to optimize resources while ensuring that advanced treatments are accessible to all patients, not just those in wealthier healthcare systems. This effort is precious for bridging the gap between innovation and equality in global healthcare.

It is also true that some interventions cannot be applied in clinical practice because clinical implementation (i.e., applicability) requires cultural and organizational change. Resistance to adopting these technologies remains, indeed, a substantial hurdle to the adoption of robotic technologies. Many healthcare professionals are hesitant to embrace new tools due to concerns about their safety, reliability, and impact on patient outcomes. This reluctance is often compounded by a lack of awareness and training. To overcome this, comprehensive education and training programs are essential. These programs should not only teach the technical aspects of using robotic devices but also emphasize their clinical benefits, ensuring that healthcare professionals are equipped to integrate these technologies into their practice effectively. Additionally, interdisciplinary collaboration—between clinicians, engineers, and policymakers—can facilitate smoother transitions from research to clinical application. Fit4MedRob aims to directly address these aspects through targeted initiatives. By fostering interdisciplinary collaboration, the program creates dedicated training frameworks that combine technical proficiency with clinical expertise. Therapists must have full awareness of the opportunities that a device can provide to the patient’s rehabilitation process, as well as of any risks linked to improper use of the device itself. Moreover, he/she must have confidence in the technology. In this perspective, the Initiative already set up Centers of Excellence that could represent an efficient solution for training healthcare operators coming from less-experienced centers, who could receive a high-level, homogenous education on new technologies. Healthcare professionals will be trained not only in the operation of robotic devices but also in understanding their full potential within patient care. Fit4MedRob will also promote continuous professional development and knowledge sharing among practitioners, which will help overcome resistance to adopting these technologies. Through pragmatic trials and the direct involvement of healthcare personnel, Fit4MedRob ensures that these new technologies are thoroughly tested in real-world environments, hopefully providing clear evidence of their benefits to both reluctant practitioners and policymakers.

From a regulatory perspective, the complexity of approving and reimbursing robotic rehabilitation technologies further complicates their clinical uptake. The evolving nature of regulatory frameworks, coupled with the absence of specific guidelines for the use of these technologies in rehabilitation, creates significant barriers that can be overcome, creating the evidence needed to close the regulatory gaps. As seen in countries like Italy, where robotic rehabilitation is already recognized as part of essential care, there is still a lack of clear reimbursement policies. A shift from a “therapist time” to a “therapy time” reimbursement model would be required to facilitate the adoption of sustainable robotic rehabilitation organizational models in which one therapist can supervise multiple patients simultaneously. Establishing robust regulatory frameworks informed by the outcomes of pragmatic trials, like those envisioned by the Fit4MedRob Initiative, will be crucial in ensuring equitable access to these advanced technologies.

The ethical and legal considerations related to patient data management also require careful attention. Robotic and digital rehabilitation technologies often involve the collection and processing of sensitive patient data, necessitating stringent data protection measures. Fit4MedRob partners are fully aware of the ethical and legal challenges associated with data management and have thus incorporated comprehensive strategies to address these issues with the aim of turning roadblockers into opportunities. The project will implement GDPR-compliant protocols that safeguard patient data at all stages—from collection to analysis and storage. Compliance with regulations such as the GDPR is essential to maintain patient trust and ensure the secure handling of personal health information as assets for research and in general for the data economy. In collaboration with legal experts and data protection officers, Fit4MedRob developed robust frameworks to prevent unauthorized access to sensitive information. Moreover, transparent consent procedures and clear communication about data usage must be integral parts of implementing these technologies. The Initiative will prioritize transparency by ensuring that all patients involved in trials are fully informed about how their data will be used, stored, and shared. Clear consent forms will be provided, and continuous monitoring will be in place to guarantee compliance with relevant data privacy regulations. These measures aim to build trust between patients, clinicians, and researchers, ensuring that technological advancements in rehabilitation are implemented responsibly and ethically.

In conclusion, while the potential of robotic and digital technologies to revolutionize rehabilitation is clear, several barriers must be addressed to achieve their full integration into clinical practice. The FIT4MedRob Initiative provides a promising framework for overcoming these obstacles through large-scale pragmatic trials, innovative organizational models, a focus on interdisciplinary training and collaboration, and on transparent and ethical approach to care. By addressing the scientific, economic, cultural, and regulatory challenges, we can move closer to a future where robotic rehabilitation is not only feasible but accessible to all patients in need. Such advancements hold the promise of significantly improving patient outcomes, optimizing healthcare resources, and shaping the future of rehabilitation practices.

## Data Availability

The original contributions presented in the study are included in the article, further inquiries can be directed to the corresponding author.

## Footnotes

1
https://www.swissneurorehab.ch/

## Group members of Fit4Mission1 group

The authors acknowledge collaborators Marco Germanotta^1^, Giovanna Nicora^2^, Alessio Fasano^1^, Maria Cristina Mauro^1^, Francesca Falchini^1^, Elena Beani^3,4^, Francesca Buganè^5^, Francesca Gennari^6^, Valerio Gower^7^, Jacopo Vitale^8^, Leopoldo Trieste^6^. 1 IRCCS Fondazione Don Carlo Gnocchi, ONLUS, Florence, Italy; 2 Department of Electrical, Computer and Biomedical Engineering, University of Pavia, Pavia, Italy; 3 Department of Developmental Neuroscience, IRCCS Fondazione Stella Maris, Pisa, Italy; 4 Department of Clinical and Experimental Medicine, University of Pisa, Pisa, Italy; 5 Research and Training Area of the INAIL Prosthetics, Centro Protesi INAIL, Istituto Nazionale Assicurazione contro gli Infortuni sul Lavoro, Bologna, Italy; 6 Scuola Superiore Sant'Anna, Pisa, Italy; 7 IRCCS Fondazione Don Carlo Gnocchi, ONLUS, Milan, Italy; 8 School of Engineering, Campus Biomedico of Rome, Rome, Italy.
